# Diffused Alveolar Hemorrhage in the Setting of Scleroderma Renal Crisis

**DOI:** 10.7759/cureus.4932

**Published:** 2019-06-18

**Authors:** Jose Henao, Raynieri Fernandez, Karla Tejada Arias, Chu Chae

**Affiliations:** 1 Internal Medicine, Advocate Illinois Masonic Medical Center, Chicago, USA; 2 Critical Care, Advocate Illinois Masonic Medical Center, Chicago, USA

**Keywords:** scleroderma renal crisis, systemic sclerosis, diffuse alveolar hemorrhage, glucocorticoids, angiotensin-converting enzyme inhibitor

## Abstract

Systemic sclerosis (SS) is a chronic, connective tissue disorder that can affect the skin, subcutaneous tissues, and internal organs. There are two different categories of SS, limited cutaneous systemic sclerosis (LCSS) and diffuse cutaneous systemic sclerosis (DCSS). One of the most fearful situations faced in DCSS is scleroderma renal crisis (SRC). This is a rare but potentially life-threatening complication characterized by an acute, usually symptomatic, increase in blood pressure, rise in serum creatinine levels, oliguria, and thrombotic microangiopathic changes. Pulmonary involvement in the setting of SCR is an even more rare combination and usually can progress into acute hypoxic respiratory failure and lead to worse outcomes. We present herein a case of scleroderma renal crisis complicated with diffuse pulmonary hemorrhage.

## Introduction

Systemic sclerosis (SS) is a chronic, connective tissue disorder that can affect the skin, subcutaneous tissues, and internal organs (most frequently, the lungs, heart, gastrointestinal tract, and kidneys) [1]. It typically presents with Raynaud phenomenon in addition to specific scleroderma-specific antibodies (anti-topoisomerase (anti-SCL-70)) and systemic manifestations depending on the related end-organ damage [2-3].

There are two different categories of SS, limited cutaneous systemic sclerosis (LCSS), which is a clinical subset of skin fibrosis seen on distal extremities and the face; and diffuse cutaneous systemic sclerosis (DCSS), which affects the skin of proximal extremities and the trunk in addition to areas affected in limited cutaneous form. This later also usually progresses more rapidly and represents a poor prognosis. Different complications could be related to this connective tissue disorder; the most prevalent are interstitial lung disease, scleroderma renal crisis, and tendon friction rubs usually seen more common in DCSS [4].

One of the most fearful situations faced in DCSS is scleroderma renal crisis (SRC). This is a rare but potentially life-threatening complication, most frequent in the first three years of the disease diagnosis with an incidence fluctuating between 5% and 10% [2,5-6]. Pulmonary involvement in the setting of SCR, also known as a pulmonary-renal syndrome, is an uncommon complication that can progress into acute hypoxic respiratory failure and lead to worse outcomes. We present herein a case of scleroderma renal crisis complicated with diffuse pulmonary hemorrhage.

## Case presentation

A 54-year-old female, with a past medical history significant for a recent diagnosis of diffuse cutaneous systemic sclerosis, hypertension, and chronic kidney disease stage three, and a history of adrenal insufficiency presented to the emergency room due to generalized weakness. The patient reported that her weakness started in the lower extremities and had been progressively worsening with difficulty both standing up and sitting. She was unable to walk for the past week. She also reported unintentional weight loss of 70 pounds for the past nine months, inadequate oral intake, decreased urine output, accompanied by dry eyes and mouth, with erythematous non-pruritic and non-painful rash in the inner thighs and buttocks.

**Figure 1:**
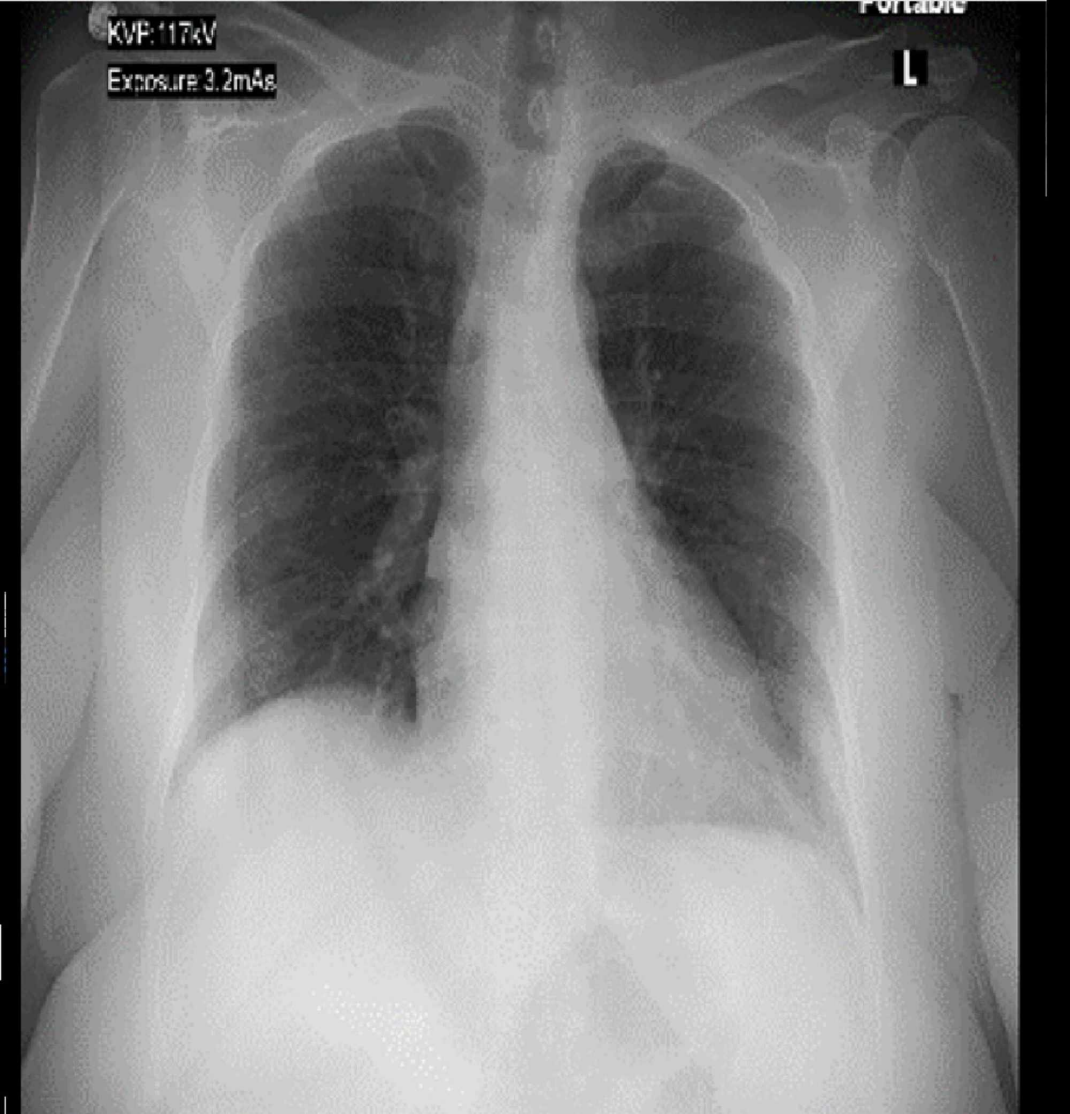
Initial X-ray Normal heart size, no mediastinal widening, no lung consolidation or effusion.

On admission, vital signs were only significant for a blood pressure of 160/80 mmHg. The physical examination was remarkable for loss of wrinkles, maculopapular rash, bilateral lower extremity with pitting edema up to the ankles, and skin breakdown in the inner thighs and sacral area. Neurological examination was normal except for decreased motor strength 3/5 in the upper extremities and 2/5 in the lower extremities. Laboratory work-up was significant for a hemoglobin level of 10.8 g/dl (normal 12-15.5 g/dl), potassium 5.8 mmol/L (normal 3.4-5.1 mmol/L), magnesium 1.5 mg/dl (normal 1.7-2.4 mg/dl), bicarbonate 14 mmol/L (normal 21-32 mmol/L), worsening creatinine 6.83 mg/dl (normal 0.51-0.95 mg/dl) (baseline creatinine 1.8-2.1 mg/dl, six months ago), and blood urea nitrogen (BUN) 107 mg/dl (normal 6-20 mg/dl). An electrocardiogram (EKG) showed a normal sinus rhythm with no other changes and her chest X-ray showed no abnormalities (Figure 1). The initial treatment consisted of treatment for hyperkalemia with regular insulin 10 units, dextrose, and polystyrene sulfonate. The patient was admitted for further workup of kidney dysfunction and weakness. The rheumatology team recommended immunologic workup, kidney biopsy, and intravenous fluid resuscitation.

**Figure 2:**
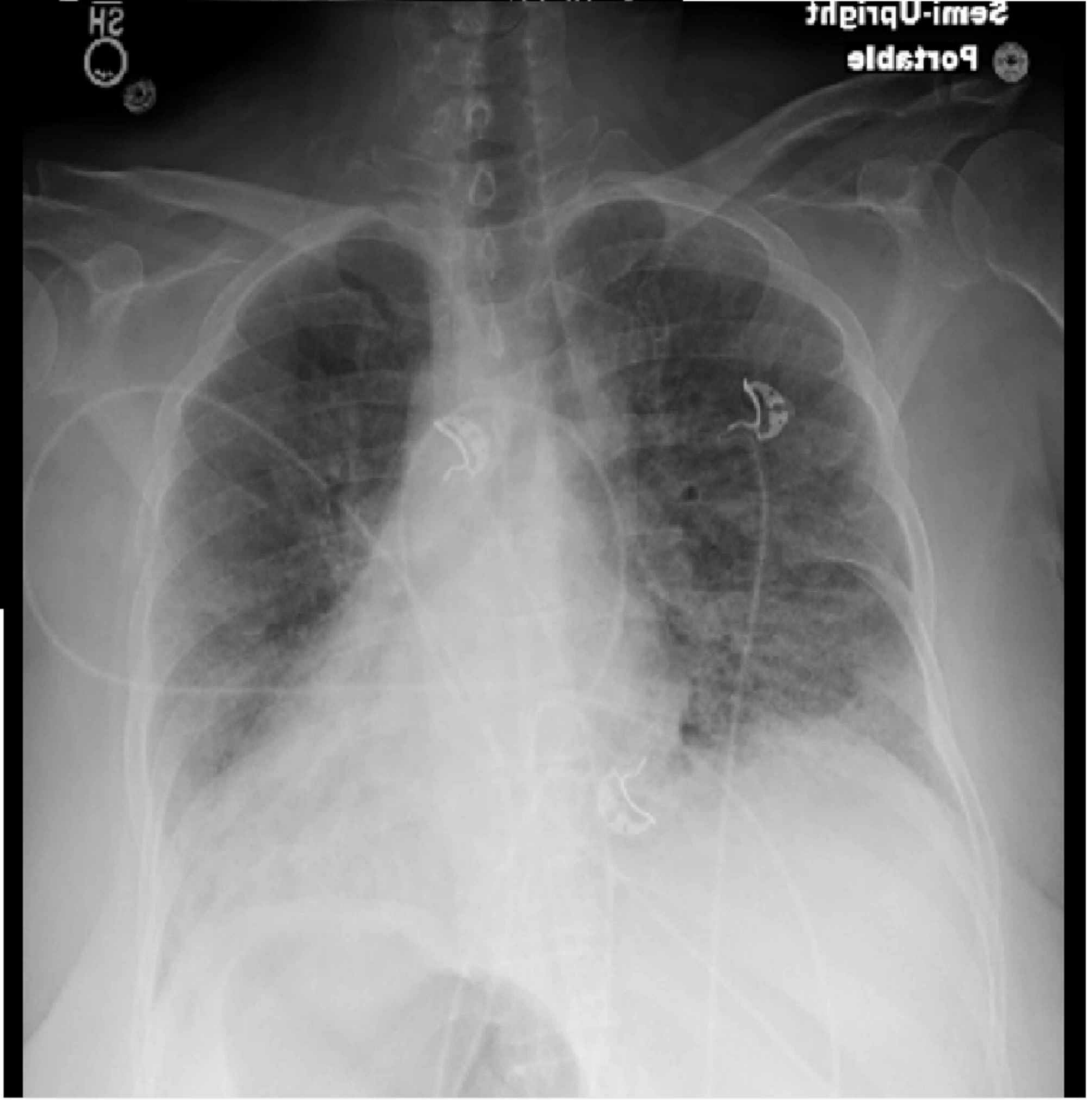
X-ray of the second day Hazy/patchy and interstitial opacity bilaterally overall increased could represent edema and/or infection, no large effusion or pneumothorax

During the second day of hospital admission, the patient started to complain of sudden onset chest pain and shortness of breath. Her vital signs were significant for blood pressure 170/90 mmHg, 115 beats per minute, and tachypnea with an oxygen saturation of 89%. The patient was placed on 4 liters of oxygen via a nasal cannula. Her physical examination was remarkable for non-reproducible substernal chest pain, right lower lobe crackles, diffused wheezing throughout, and marked pitting edema in bilateral lower extremities up to the knee. Intravenous fluids were stopped, and a trial of albuterol inhaler was given, with no improvement. As part of the initial workup, the laboratory was remarkable for elevated troponin 0.07 ng/ml (normal <0.05 ng/ml), d-dimer 2.5 mg/l (normal <0.57 mg/l) and procalcitonin 19.9 ng/ml (normal<0.10 ng/ml). A new electrocardiogram (EKG) showed sinus tachycardia with no ischemic changes, and chest X-ray showed current ground-glass densities with interstitial opacities bilaterally (Figure 2). The patient was transferred to the medical intensive care unit (MICU) for further care.

**Figure 3:**
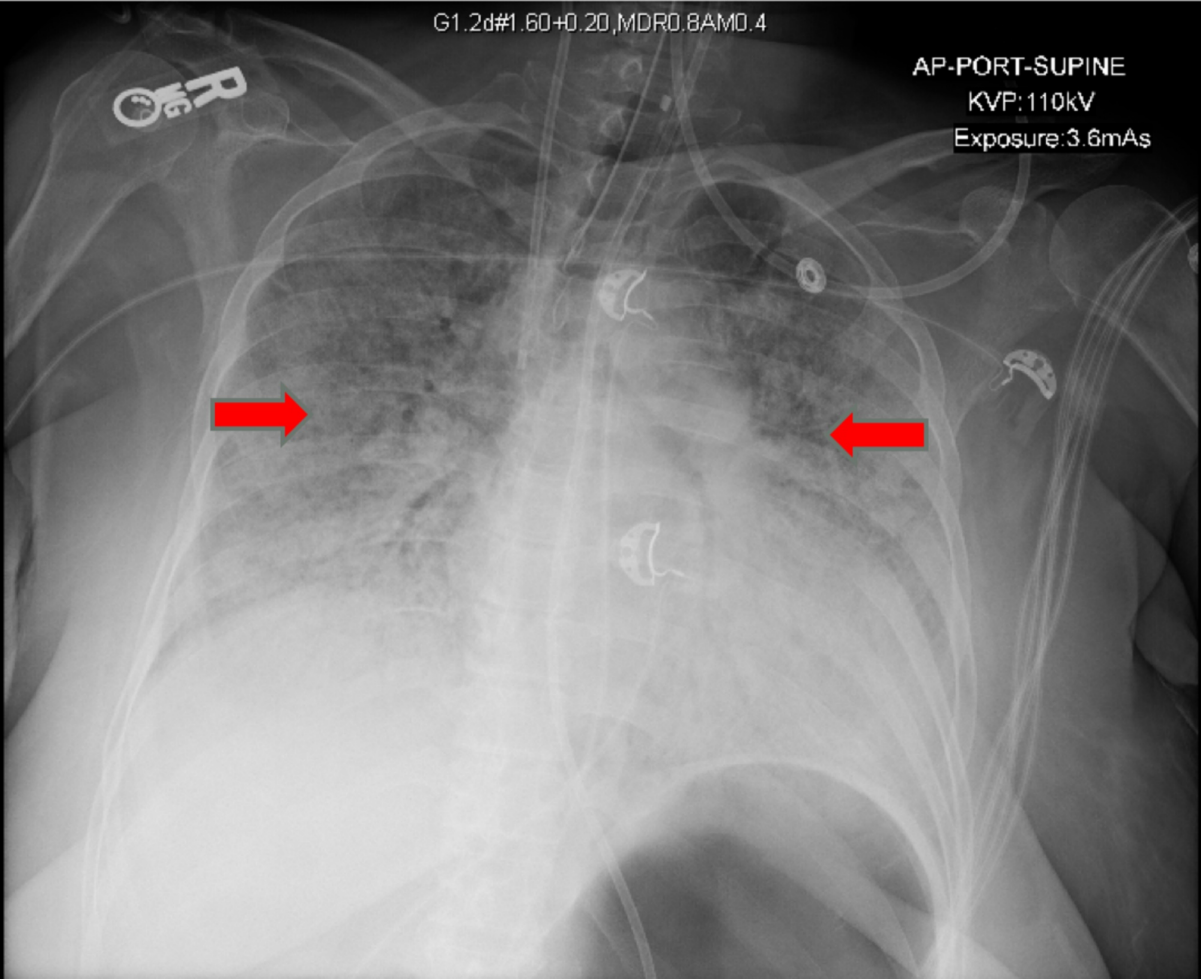
X-ray after intubation Endotracheal tube projects at the upper margin of the right clavicle, rotated, right internal jugular (IJ) temporary hemodialysis catheter in the right brachycephalic vein, nasogastric tube in place. Extensive diffuse pulmonary confluent airspace opacities.

At arrival to the MICU, she was progressively getting worse and using accessory respiratory muscles. Her arterial blood gas (ABG) showed pH 7.20 unit (normal 7.35-7.45 unit), CO_2_ 22 mmHg (normal 32-45 mmHg) and O_2_ 54 mmHg (normal 83-108 mmHg) on 6 L through a nasal cannula. The patient was transitioned to bilevel positive airway pressure (BiPAP), but no improvement was achieved. Later, ABG with worsening acidosis and CO_2_ retention (PH 7.15 and CO_2 _45), a potassium level of 6.2 mmol/L (normal 3.4-5.1 mmol/L), BUN 150 mg/dl (normal 6-20 mg/dl), and creatinine 7.9 mg/dl (normal 0.51-0.95 mg/dl). The decision was made to proceed with intubation for ventilatory support; she was started on empiric antibiotics (ceftriaxone and azithromycin), intravenous heparin for suspected pulmonary embolism, and emergent dialysis for worsening uremia. Repeated chest X-ray after intubation showed worsening bilateral diffuse interstitial opacities concerning for different etiologies, including pleural effusion, pneumonia, or pulmonary hemorrhage (Figure 3). Later during the day, a small amount of blood through the endotracheal tube (ETT) was noted, thereby intravenous heparin was held.

**Figure 4:**
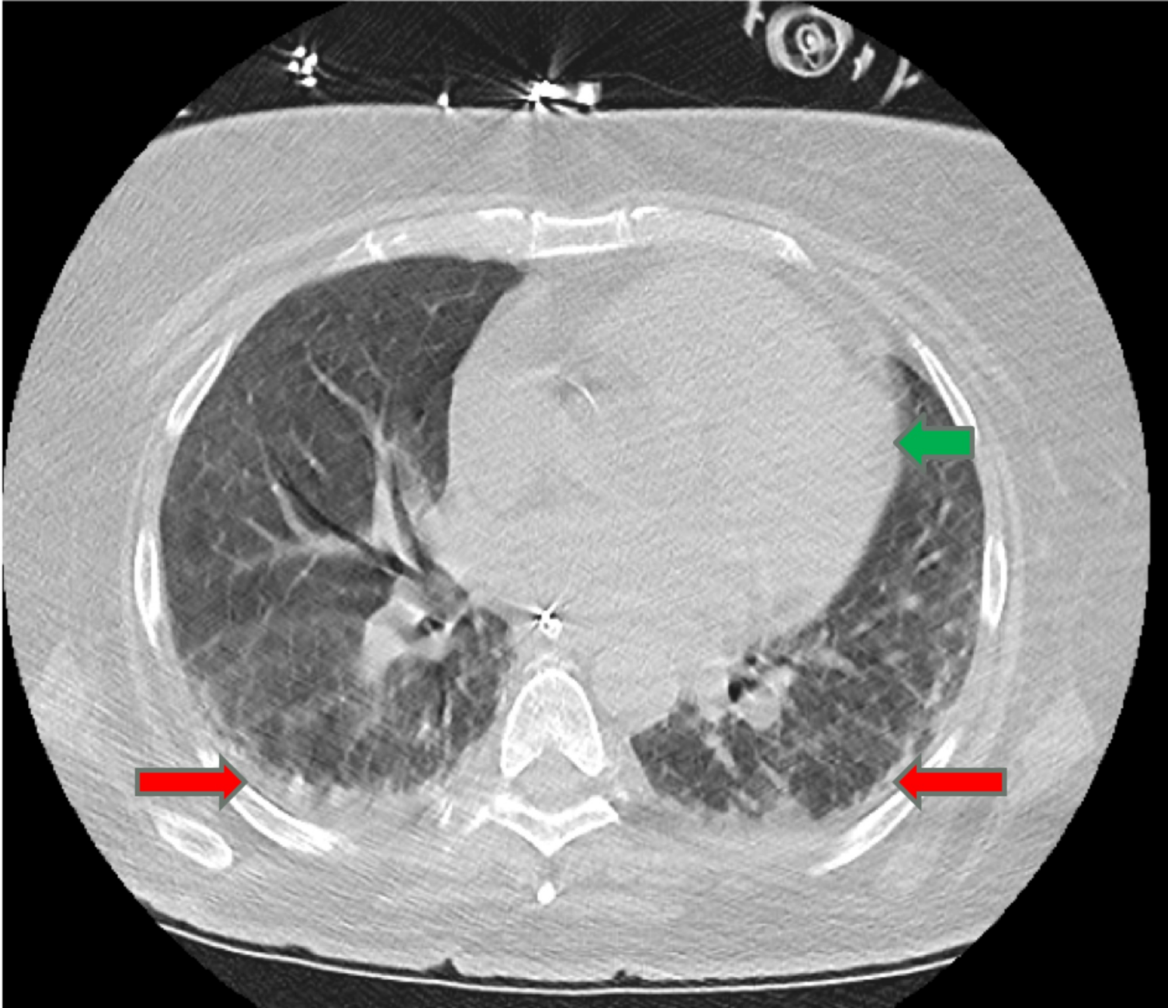
Chest CT Bibasilar atelectatic changes (red arrow), no gross evidence of interstitial lung disease. Cardiomegaly (green arrow).

At this point, owing to the findings of worsening kidney function, fluid overload, and persistent hypertension with systolic blood pressure >200/100 mmHg, the primary diagnosis was SRC. Management includes starting the patient on angiotensin-converting enzyme (ACE) inhibitor. Further extensive investigations only showed lower levels of decreased hemoglobin 7.7 g/dl (normal 12-15.5 g/dl), low complement C3 40mg/dl (normal 79-152mg/dl), and high lactate dehydrogenase (LDH) levels of 625 U/L (normal 82-240 U/L). The peripheral smear showed mildly hypochromic anemia and rare polychromatophilic cells. Imaging included transthoracic echocardiogram that revealed concentric hypertrophy, ejection fraction 50%-55%, and grade one diastolic dysfunction. Bilateral lower extremities ultrasound gave no evidence of deep venous thrombosis. Computed tomography of the chest showed bibasilar atelectatic changes, no gross evidence of interstitial lung disease, and cardiomegaly (Figure 4).

**Figure 5:**
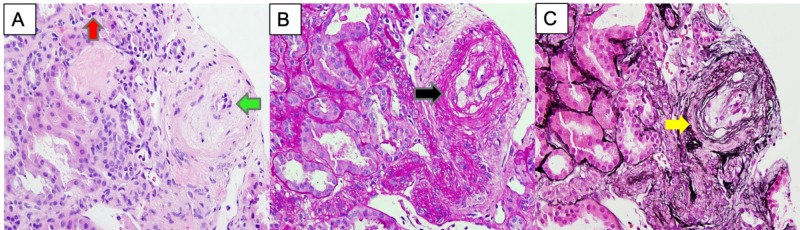
Pathology of the glomerulus A. (High-power magnification; H&E) Severe arteriolar mucoid intimal thickening showing a considerable reduction of the arterial lumen with adjacent sclerotic changes (green arrow). Schistocytes and several fibrin thrombi can be appreciated within the lumen of small adjacent vessels (red arrow). B. (PAS stain; high- power magnification) Arteriolar thickening with adjacent tubular epithelium showing loss of brush border and tubular epithelial damage(black arrow). C. (Jones silver stain; high- power magnification) Arteriolar intimal damage (yellow arrow).

During the fourth day of hospitalization, the patient's condition worsened, with persistent moderate to large amounts of blood through the ETT with a significant drop in the hemoglobin to 5.7 g/dl (normal 12-15.5 g/dl) from which she received a unit of packed red blood cells. Bronchoscopy was later done, revealing moderate thick bloody secretions in the tracheobronchial tree consistent with pulmonary hemorrhage. Intravenous steroids were held to avoid the worsening of SRC. The kidney biopsy reported an acute thrombotic microangiopathy type pattern with severe vascular involvement, most consistent with scleroderma renal crisis associated with tubular necrosis (Figure 5).

Ultimately, the patient was transferred to a tertiary scleroderma center, in which she significantly improved after supportive treatment and infusion with cyclophosphamide. The patient was successfully extubated and was transitioned to acute inpatient rehabilitation.

## Discussion

SRC is characterized by an acute, usually symptomatic, increase in blood pressure, rise in serum creatinine levels, oliguria, and thrombotic microangiopathic changes [5]. There are numerous risk factors that have been identified, most commonly diffuse skin involvement, presence of auto-antibodies, glucocorticoid, and cyclosporine use [7-9]. In our case, it was a typical presentation only identified as a potential risk of diffuse skin involvement, which is an independent predictor for rapid progression.

The European League Against Rheumatism (ELAR) recommends using angiotensin-converting-enzyme inhibitor (ACE) inhibitors to treat scleroderma renal crisis since they are associated [3]. ACE inhibitors are approved for patients with SRC and associated with improved survival [10-12]. Other alternatives include calcium channel blocker, angiotensin receptor blockers, and alpha-blockers [7]. Dialysis may be required in patients with scleroderma renal crisis. The current survival of patients with SCR is 70%-82% at one year but decreases to 50%-60% at five years despite dialysis support. The patients who show no signs of renal functional recovery despite timely blood pressure control are candidates for transplantation [8]. As shown in the case, therapy with ACE inhibitors significantly improved the patient’s overall condition in relationship to hypertension even though it did not prevent hemodialysis.

SS is associated with multiple visceral complications; pulmonary involvement is the second most frequent, preceded by esophageal dysfunction. Even though the most frequent pulmonary complications are interstitial lung disease (ILD) and pulmonary artery hypertension (PAH), there is a specific infrequent group that can present as diffuse alveolar hemorrhage (DAH). The unique characteristics of SS may alter the approach to alveolar hemorrhage, in this particular population steroids, although it could be beneficial for treatment, should be used cautiously since can precipitate SRC [13-15].

Acute hypoxemic respiratory failure is severe arterial hypoxemia that is refractory to supplemental oxygen. It can be caused by elevated alveolar capillary hydrostatic pressure, increased alveolar-capillary permeability, and exudates. The differential diagnosis included pulmonary-renal syndrome (PRS) was considered. PRS is an entity described as an acute renal failure with diffuse alveolar hemorrhage. Prompt differential diagnosis between the subsets is critical because therapeutic strategy may differ in the use of high-dose corticosteroid and plasma [16]. This diagnosis was ruled out with a biopsy.

## Conclusions

High suspicion is needed for the early recognition of potential complications in patients with respiratory distress in the setting of scleroderma renal crisis. Starting treatment in a timely fashion is essential. Management with glucocorticoids should be cautious since it could precipitate worsening renal function.

## References

[1] Denton CP (2015). Advances in pathogenesis and treatment of systemic sclerosis. Clin Med Lond Engl.

[2] Hinchcliff M, Varga J (2008). Systemic sclerosis/scleroderma: a treatable multisystem disease. Am Fam Physician.

[3] Kowal-Bielecka O, Fransen J, Avouac J (2017). Update of EULAR recommendations for the treatment of systemic sclerosis. Ann Rheum Dis.

[4] Denton CP, Khanna D (2017). Systemic sclerosis. Lancet Lond Engl.

[5] Klein-Weigel P, Opitz C, Riemekasten G (2011). Systemic sclerosis - a systematic overview: part 1 - disease characteristics and classification, pathophysiologic concepts, and recommendations for diagnosis and surveillance [Article in German]. Vasa.

[6] Woodworth TG, Suliman YA, Li W, Furst DE, Clements P (2016). Scleroderma renal crisis and renal involvement in systemic sclerosis. Nat Rev Nephrol.

[7] Denton CP, Lapadula G, Mouthon L, Müller-Ladner U (2009). Renal complications and scleroderma renal crisis. Rheumatol Oxf Engl.

[8] Muangchan C, Canadian Scleroderma Research Group (2013). The 15% rule in scleroderma: the frequency of severe organ complications in systemic sclerosis. A systematic review. J Rheumatol.

[9] Bose N, Chiesa-Vottero A, Chatterjee S (2015). Scleroderma renal crisis. Semin Arthritis Rheum.

[10] Steen VD, Costantino JP, Shapiro AP, Medsger TA Jr (1990). Outcome of renal crisis in systemic sclerosis: relation to availability of angiotensin converting enzyme (ACE) inhibitors. Ann Intern Med.

[11] Steen VD, Medsger TA (2000). Long-term outcomes of scleroderma renal crisis. Ann Intern Med.

[12] Walker KM, Pope J (2012). Treatment of systemic sclerosis complications: what to use when first-line treatment fails—a consensus of systemic sclerosis experts. Semin Arthritis Rheum.

[13] Colaco B, Khasawneh K (2013). Diffuse alveolar hemorrhage in the setting of scleroderma renal crisis. Chest.

[14] Herndon TM, Kim TT, Goeckeritz BE, Moores L, Oglesby R, Dennis G (2001). Alveolar hemorrhage and pulmonary hypertension in systemic sclerosis: a continuum of scleroderma renal crisis?. J Clin Rheumatol Pract Rep Rheum Musculoskelet Dis.

[15] Chaer RA, Massad MG, Evans A, Olopade C, Varga J (2001). Systemic sclerosis complicated by diffuse alveolar hemorrhage. Med Sci Monit Int Med J Exp Clin Res.

[16] Naniwa T, Banno S, Sugiura Y (2007). Pulmonary-renal syndrome in systemic sclerosis: a report of three cases and review of the literature. Mod Rheumatol.

